# On the use of bolus for pacemaker dose measurement and reduction in radiation therapy

**DOI:** 10.1002/acm2.12229

**Published:** 2017-11-20

**Authors:** Huagang Yan, Fanqing Guo, Dengsong Zhu, Stefan Stryker, Sharron Trumpore, Kenneth Roberts, Susan Higgins, Ravinder Nath, Zhe Chen, Wu Liu

**Affiliations:** ^1^ Department of Therapeutic Radiology Yale University School of Medicine Yale‐New Haven Hospital New Haven CT USA; ^2^ School of Biomedical Engineering Capital Medical University Beijing China; ^3^ Department of Radiation oncology Saint Thomas Hospital Murfreesboro TN USA; ^4^ Western Kentucky University Bowling Green KY USA

**Keywords:** bolus, cardiac implantable electronic device (CIED), defibrillator, out‐of‐field dose, pacemaker, radiation therapy, treatment planning system

## Abstract

Special attention is required in planning and administering radiation therapy to patients with cardiac implantable electronic devices (CIEDs), such as pacemaker and defibrillator. The range of dose to CIEDs that can induce malfunction is large among CIEDs. Clinically significant defects have been reported at dose as low as 0.15 Gy. Therefore, accurate estimation of dose to CIED and dose reduction are both important even if the dose is expected to be less than the often‐used 2‐Gy limit. We investigated the use of bolus in in vivo dosimetry for CIEDs. Solid water phantom measurements of out‐of‐field dose for a 6‐MV beam were performed using parallel plate chamber with and without 1‐ to 2‐cm bolus covering the chamber. In vivo dosimetry at skin surface above the CIED was performed with and without bolus covering the CIED for three patients with the CIED <5 cm from the field edge. Chamber measured dose at depth ~0.5–1.5 cm below the skin surface, where the CIED is normally located, was reduced by ~7–48% with bolus. The dose reduction became smaller at deeper depths and with smaller field size. In vivo dosimetry at skin surface also indicated ~20%–60% lower dose when using bolus for the three patients. The dose measured with bolus more accurately reflects the dose to CIED and is less affected by contaminant electrons and linac head scatter. In general, the treatment planning system (TPS) calculation underestimated the dose to CIED, but it predicts the CIED dose more accurately when bolus is used. We recommend the use of 1‐ to 2‐cm bolus to cover the CIED during in vivo CIED dose measurements for more accurate CIED dose estimation. If the CIED is placed <2 cm in depth and its dose is mainly from anterior beams, we recommend using the bolus during the entire course of radiation delivery to reduce the dose to CIED.

**Abbreviations**
AAA: Analytical Anisotropic Algorithm
AAPM: The American Association of Physicists in Medicine
CIED: cardiac implantable electronic device
OSLD: optically stimulated luminescence detector
SSD: source‐to‐surface distance
TPS: treatment planning system

## 1 INTRODUCTION

As the average life span of the world population grows, there is also an increase in the use of cardiac implantable electronic devices (CIEDs), including pacemakers and defibrillators, to maintain normal cardiac function in aged humans. This increased CIED use can be seen in data that show the use of pacemakers growing by 55.6% from 1993 to 2009.^1^ These devices prove beneficial to cardiac health, but they add limitations to radiation therapy given the pulse generator's inability to tolerate certain levels of radiation dose. The likelihoods of having a dependency on a CIED and contracting cancer are both tied to aging.^2^ The dose limitation added by the presence of a pacemaker near a tumor during radiation treatment is a growing problem. There is a need to find innovative and simple ways to lower and more accurately estimate the dose delivered to the CIEDs.

Mouton et al^3^ observed that varying levels of dose can cause parasitic signal, alter the properties of semiconductor materials, and even cause permanent silence within CIEDs. Some of these problems are temporary, i.e., after radiation treatment, the CIED can return to its normal operation. The permanent silence failure causes the pulse generator to permanently cease operation. AAPM TG34 report^4^ recommends the total accumulative dose received by pacemakers to be less than 2 Gy. Since the publication of AAPM TG34 report, cardioverter defibrillators have also been widely used. Medtronic provides 1–5 Gy dose limits to their defibrillators for different models. Other vendors did not provide an explicit safe limit for their devices. Therefore, many clinics limit the defibrillator dose to less than 1 Gy in the absence of a specific recommendation from device manufacturer. However, Mouton and colleagues^3^ observed that clinically important defects could occur at doses as low as 15 cGy and faults that cause discomfort could occur at doses as low as 5 cGy. They noted that even temporary CIED failures, triggered at lower dose, could create health risks. The more recently published Netherland guideline^5^ gives extra attention to pacing dependent patients. Even if the CIED dose is less than 2 Gy, those patients are still considered at medium risk instead of low because CIED malfunction is especially deleterious; therefore, the guidelines for medium risk should be followed.^5^

With technology improvements, the average size of pacemakers has decreased. This has caused pacemakers to become more susceptible to damage caused by radiation.^6^ The “increased circuit complexity, the ever‐decreasing power consumption, and possibly the decreased dose attenuation of the CIED case” also contribute to the radiosensitivity.^5^ It has been recommended to know specific pacemaker make and model and the manufacturer's guidelines when planning radiation treatment near patient pacemakers.^7^ Hurkmans et al^5^ provided a comprehensive review on the topic of managing radiation oncology patients with CIEDs. We will briefly summarize some new papers after the publication of that review.

Treatment plans involving patients with CIEDs should keep the pacemaker out of the direct beam^4^ and use less than 10‐MV beams.^8^ Many clinics, including ours, consider 6 MV as optimal for CIED patients and eliminate the use of high photon energy for those patients. This is because neutrons produced in high‐energy beams are believed to be the main cause of CIED defects.^5^ The dose to CIED lowers as the distance to the radiation fields increases. It is a challenge to accurately calculate the dose to the CIEDs though because clinical dose calculation algorithms are less accurate for out‐of‐field dose.^8^ Several more accurate empirical dose prediction methods have been developed for out‐of‐field dose.^8^, ^9^ However, they are not conveniently available for everyone and still have more than 10%–20% errors even for the better cases. This lower accuracy supports the use of thermoluminescent dosimeters (TLDs) or optically stimulated luminescence detectors (OSLDs) to measure the dose in the CIED region.^5^, ^7^ In this region, there could be a large discrepancy between the measured dose and the dose predicted by the treatment planning systems (TPSs).^8^

For certain cases, some specialized clinics will relocate the CIED to lower its dose, which is complicated and invasive.^5^ Hurkmans et al^5^ reported that the risk of relocation is comparable to the potential risk for CIED problems related to radiotherapy. Bourgouin et al^9^ studied the impact of dose reduction using lead wrapped in thermoplastic to shield CIEDs from therapeutic radiation. The study simulated treatment of 6 and 23 MV beams by using the shielding on solid water phantom and found an average dose reduction of 19 ± 13%, and the reduction for 23 MV beams was more substantial than 6 MV beams. TPSs are often unable to properly calculate dose in the presence of lead foil,^9^ which makes dose estimation difficult. We propose a simpler method by covering the CIED with 1‐ to 2‐cm bolus (such as Superflab^TM^) during radiation treatment, which should reduce the dose to CIEDs from anterior beams. For posterior beams, backscatter from the bolus to pacemaker should be less than that from the high‐Z lead foil. When estimating dose to pacemaker, the dose measured by dosimeters under bolus should reflect more accurately the pacemaker dose compared to placing the dosimeters on the skin surface without covering it with bolus. We conducted phantom and patient measurements with and without bolus and using bolus only and bolus covered with lead to test this method.

## 2 MATERIALS AND METHODS

**Figure 1:**
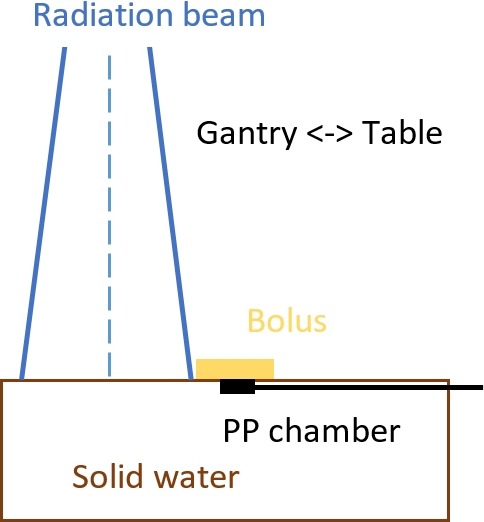
Schematic illustration of the “out‐of‐field” dose measurement in solid water phantom.

Out‐of‐field dose was measured with a Markus parallel plate chamber at depths varying by 0.5‐cm intervals between 0 and 3 cm in a solid water phantom for a 6 MV beam (Varian 2300IX). For each of these depths, measurements were conducted at distances of 2, 3, and 5 cm from the beam field edge. Figure 1 illustrates the experimental setup of the phantom measurements. A parallel plate chamber was used because it is suitable for measuring dose at surface/build‐up region in photon beams due to their thin entrance window and depth positioning accuracy.^10^ The treatment plan was one AP field set to deliver 300 cGy at *d*_max_ with 100 cm source‐to‐surface distance (SSD). Measurements were performed with and without bolus at three field sizes (5 × 5 cm, 10 × 10 cm, and 15 × 15 cm) and two collimator angles (0° and 90°). For 10 × 10 cm field and collimator 90°, measurements were performed without bolus, with 1‐cm bolus, with 2 mm lead on top of 1‐cm bolus, and with 2‐cm bolus covering the chamber. These measurements were repeated on three different days to evaluate measurement uncertainty. To establish the conversion of ionization to dose, 100 MU was delivered at 100 SSD, 10 × 10 cm field with the chamber placed at *d*_max_ in the solid water phantom. The dose in this condition is 100 cGy based on machine calibration. Chamber reading was converted to dose accordingly. Eclipse Analytical Anisotropic Algorithm (AAA) v13.6 was used to calculate dose values to be compared with the measured dose. The calculated dose is a volume average of a structure with 5.3 mm diameter on a single 2‐mm CT slice, which corresponds to the Markus chamber dimension.

**Figure 2:**
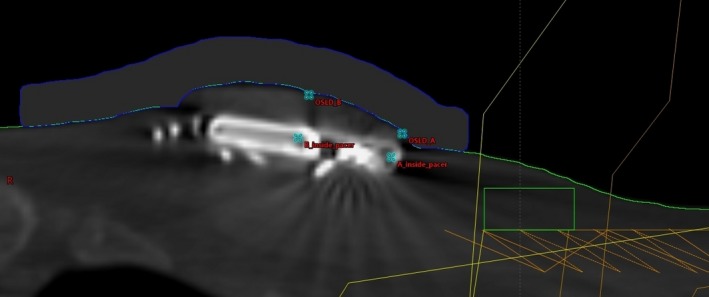
Setup of the *in vivo* measurements of the pacemaker dose. The blue contour shows 1‐cm bolus. The CIED is about 1 cm below the skin surface.

*In vivo* dose measurements were also conducted for three patients treated with 3D conformal 6 MV beams using OSLDs (nanoDot^TM^, Landauer) placed on the skin above the CIED. Two beams were used for two patients and three for the other patient. Beam directions are listed in Table 1. According to the manufacturer, the accuracy of screened nanoDot is ±5.5%. Trials were performed on different treatment days in order to measure doses without bolus and with 1‐ or 2‐cm bolus covering the OSLDs. With bolus, the measurements were conducted with and without 2 mm lead foil on top of the bolus. The measurement situations were slightly different for each patient as listed in Table 1. The pacemakers were within 5 cm to the closest treatment field edge. Figure 2 shows the setup of patient measurements. Two OSLDs were placed on the skin surface, one at a location above the pacemaker near to the closest anterior treatment field edge (OSLD_A in Fig. 2), and the other one approximately above the center or the distal portion of the pacemaker (OSLD_B in Fig. 2). The doses are calculated using Eclipse AAA algorithm with and without bolus at four locations: OSLD_A, OSLD_B, A‐inside_pacer (close to OSLD_A but at a depth inside the pacemaker), and B_inside_pacer.

**Table 1:** Measured and calculated dose data for different patients

		Patient 1			Patient 2			Patient 3		
Beam directions		RAO and LPO			AP, LAO, and RPO			RAO and LAO		
Bolus thickness on skin		0 cm	1 cm	1 cm + lead	0 cm	1 cm	1 cm + lead	0 cm	2 cm	2 cm + lead
Measured dose (cGy)	OSLD_A	52.8	n/a	40.8	163.1	74.4	72.4	48.1	21.5	19.9
	OSLD_B	27.4	n/a	21.0	103.9	45.7	41.7	34.9	13.2	12.1
Eclipse AAA calculated dose (cGy)	OSLD_A	86.2	39.8	–	323.2	89.0	–	82.0	23.5	–
	OSLD_B	42.4	22.3	–	159.4	30.6	–	71.8	9.2	–
	A_inside_pacer	46.0	43.1	–	125.9	90.5	–	29.2	25.7	–
	B_inside_pacer	27.4	26.3	–	31.3	25.4	–	13.2	9.8	–

Measured dose is listed as the average OSLD readings multiplied by the number of fractions to estimate the accumulative doses over the entire course, and plan calculated doses refer to locations defined in Fig. 2.

## 3 RESULTS AND DISCUSSION

### 3.A Phantom measurements

#### 3.A.1 Measurement at different field sizes

**Figure 3:**
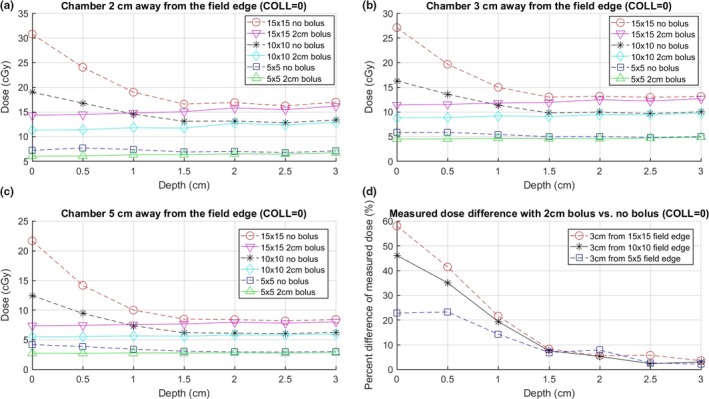
Dose at various depths and distances from the field edge for 300 MU, 100 SSD, 0° collimator, 6 MV AP beam of 5 × 5 cm, 10 × 10 cm, 15 × 15 cm field size. (a), (b), and (c) show dose at distances of 2, 3, and 5 cm from the field edge, respectively. (d) The percent difference in the measured dose 3 cm from the field edge with 2‐cm bolus vs no bolus, i.e., 100 × (dose_without − dose_with) / dose_without % of the measured doses, which shows the reduction in the measured dose with vs without bolus.

Figures 3(a)–3(c) show the chamber measured doses for solid water phantom with and without 2‐cm bolus at various distances from the beam edge and depths for 5 × 5 cm, 10 × 10 cm, and 15 × 15 cm fields (AP, collimator = 0). All chamber measurements are the average of three readings. The standard deviations of the readings are all less than 0.2%. The error bars are smaller than the symbols, hence omitted in the plots. To estimate the setup uncertainty, the measurements at 3 cm away from the field edge of 10 × 10 cm field were repeated in three different days. The average relative uncertainty (one standard deviation) is 0.5% with maximum of 1.8%. Figure 3(d) shows the percent difference in the measured dose with and without bolus, i.e., 100 × (dose_without − dose_with) / dose_without % of the measured dose, at 3 cm away from the field edge. The trend for 2 and 5 cm away is similar to that for 3 cm away. Therefore, those results are not plotted in order that the figure is clear to read.

The patient's CIED is normally at 0.5–1.5 cm below the skin surface. For that depth, adding 2‐cm bolus reduces the CIED dose by about 7–48% if the dose is mostly from anterior beams. The percent reduction depends strongly on the CIED depth, moderately on the field size, and weakly on the distance to the field edge. At deeper depths, the reductions are small. The dose reduction is more significant at larger field sizes. The shape of the bolus curves relative to the nonbolus curves shows that the use of bolus is able to eliminate the high dose in the shallow region. The benefits of using a bolus to reduce dose to a CIED diminish if the CIED is located deeper than 1.5–2 cm, as it can be seen in Fig. 3(d) that the percent dose reduction at larger depth becomes insignificant.

#### 3.A.2 Measurement at different collimator angles

**Figure 4:**
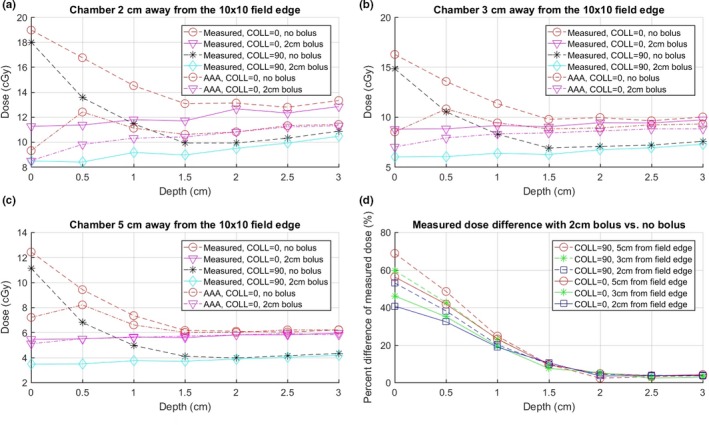
Dose at various depths and distances from the field edge for 300 MU, 10 × 10 cm, 100 SSD, 6 MV AP beam at collimator at 0° and 90°. (a), (b), and (c) show dose at distances of 2, 3, and 5 cm from the field edge, respectively. (d) The percent difference in the measured dose with 2‐cm bolus vs no bolus at different collimator angles, i.e., 100 × (dose_without − dose_with) / dose_without % of the measured doses, which indicates the reduction in the measured dose with vs without bolus.

Figure 4 shows the measured and Eclipse calculated doses with and without 2‐cm bolus for 10 × 10 cm field with collimator angles at 0° and 90° (setup as Fig. 1), as well as the percent difference in the measured dose with and without bolus. The measured out‐of‐field near surface dose is larger for 0° collimator. A reasonable explanation is that the x jaw is the lower jaw on Varian machines; therefore, at 0°, more collimator scatter can reach the chamber in the setup shown in Fig. 1. The percent reductions are slightly more for 90° collimator and when distance to the closest treatment field is increased.

**Figure 5:**
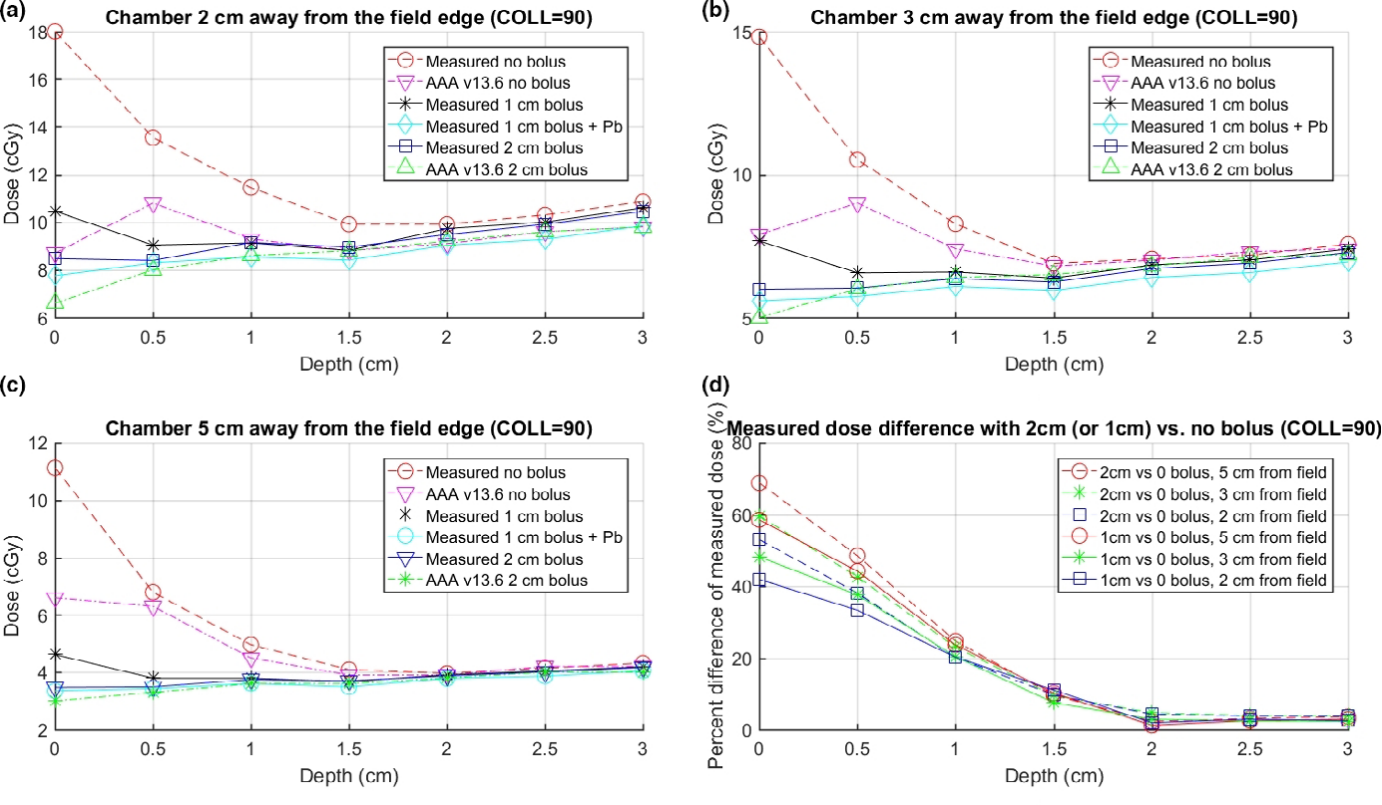
Dose at various depths and distances from the field edge under different bolus settings for 300 MU, 10 × 10 cm, 100 SSD, 90° collimator, 6 MV AP beam. (a), (b), and (c) show dose at distances of 2, 3, and 5 cm from the field edge, respectively. (d) The percent difference in the measured dose with 2 (or 1) cm bolus vs no bolus, i.e., 100 × (dose_without − dose_with) / dose_without % of the measured doses, which shows the reduction in the measured dose with vs without bolus.

Figures 4 and 5 indicate that the Eclipse AAA v13.6 algorithm tends to underestimate the out‐of‐field dose compared with the measurements (especially when the distance to the field edge is small) and does not model accurately for depths less than 1.5 cm. One possible explanation is that accurate dose modeling for regions both out‐of‐field and near the surface is difficult with the commissioning data because it probably needs more detailed information and modeling for gantry leakage and collimator scattering. To keep the readability of the figures, only AAA calculations at 0° collimator are displayed in Fig. 4. The AAA calculations at 90° collimator are shown in Fig. 5.

#### 3.A.3 Measurement with different bolus thickness

Figure 5 shows the measured doses without bolus, with 1‐cm bolus, with 2 mm lead on top of 1‐cm bolus, and with 2‐cm bolus covering the CIED and Eclipse calculated doses with and without 2‐cm bolus, as well as the percent difference in the measured dose with and without bolus. The results indicate that all three bolus configurations provide similar amount of dose reduction for the depth more than 1 cm. However, for depth less than 1 cm, using 2 cm yields slightly more dose reduction than using 1‐cm bolus.

#### 3.B Patient data

*In vivo* measurements and plan calculated results for the three patients are listed in Table 1. The measured doses in cGy are the average OSLD readings multiplied by the number of fractions to estimate the total dose over the entire course. Because the bolus shielded a significant portion of the electron contamination as well as scatter radiation from the linac head components such as MLC, the measured doses on the skin surface with bolus were substantially reduced compared to the case with no bolus. The bolus does not affect the internal scatter to the CIED. For all three patients, the percentage of dose reduction for OSLD_A is similar to that for OSLD_B. Adding a 2‐mm lead foil on top of the bolus did not provide significant further dose reduction. This can be explained because the bolus alone has already shielded most of the electron contamination and MLC scatter. The dose reduction with vs without bolus for patient 1 is about 20%. The reduction for patients 2 and 3 is about 50%–60%. The difference may be explained because, for patient 1, the contribution from the posterior beam is large and adding bolus does not shield the posterior beam, while for patient 2, the CIED dose is mostly from the anterior beams and patient 3 does not have a posterior beam.

In clinical practice, the dose to the CIED for each patient is estimated by the TPS prior to the start of treatment to determine whether *in vivo* dose measurement is needed and whether additional checkup of the CIED and ECG monitoring during treatment are needed.^4^, ^5^ It is convenient to estimate the dose using TPSs. However, TPSs are not commissioned for out‐of‐field dose calculation; therefore, the estimation accuracy is significantly limited, as can be seen from the differences between the Eclipse calculated doses and the measured doses shown in Table 1. It should be noted that, in addition to the inaccuracies in the TPS algorithm, the differences can also be attributed to the uncertainties related to the positioning of the OSLDs. Because dose estimation accuracy should be high enough to determine the potential risk that the treatment may pose, 5 we are conservative in our clinic and normally perform *in vivo* measurements unless the closest treatment field edge is very far away from the CIED.

It is worth noting that, based on Figs. 3, 4, 5, for the measurements without bolus, the dose at zero depth is markedly higher than the values in the 0.5–1.5 cm depth range where the CIED is normally located. This indicates that the measurements conducted on patients at the skin surface without bolus will inherently be an overestimation of the dose delivered to the CIED. The measured dose under bolus is approximately equivalent to the dose without bolus at a depth close to the bolus thickness, which is approximately the depth of pacemaker. Therefore, for more accurate estimation of the CIED dose, bolus should be used to cover the CIED. Modern CIEDs are often thin, and sometimes, the top portion of the CIED may be placed very shallow under the skin surface (<0.5 cm). In these cases, dose measured under a thick bolus may underestimate the maximum dose to the CIED. However, if we cover the CIED with 1‐ to 2‐cm bolus (depending on the depth of CIED and beam configuration) during the entire course of treatment, the measured dose under bolus can be used as a sufficiently accurate estimation of the dose to CIED. This is because the large uncertainty happens only near the surface, and the dose gradient is not steep at deeper regions. In addition, the bolus' shielding effect also reduces the dose to CIED. We, therefore, recommend using bolus to cover CIED at all treatment fractions unless the CT scan shows that the entire CIED is >2 cm deep in the body. In that case, Fig. 3 shows that adding bolus as shielding may not be necessary as the dosimetric difference is small. However, this is not normally seen for modern CIEDs. In addition, when performing *in vivo* dosimetry, adding bolus is still useful in estimating more accurately the actual dose to CIED.

Another important consideration is the treatment beam configuration. It is more effective to use bolus if the dose contribution from the anterior beams is much larger than that from the posterior beams. In fact, for posterior beams, adding a lead shield can slightly increase the backscatter to the CIED.^9^ Backscatter can also be expected for bolus, but to a lesser extent compared to the high‐Z material.

From both Figs. 4 and 5 and Table 1, it is noticed that the TPS calculated doses are reasonably in agreement with the measured dose with the presence of bolus. For example, this can be seen from Table 1 by comparing the measured dose under bolus to the calculated dose inside the CIED. It suggests that the use of a bolus on patients can not only serve to lower the dose to the CIED, but also prove to be beneficial to allow the use of the TPS to more accurately estimate the CIED dose prior to treatment.

## 4 CONCLUSIONS

*In vivo* CIED dose measurements with the bolus covering dosimeter on the skin above the CIED reflect more accurately the delivered dose to the CIED. Phantom measurements and patient *in vivo* dosimetry show that covering the CIED with 1‐ to 2‐cm bolus during radiotherapy can substantially reduce the dose to CIED if the CIED is implanted at very shallow depths (<2 cm below skin surface) and most CIED dose is from anterior radiation beams. Adding the bolus also helps the TPS to more accurately predict the CIED dose prior to treatment for well‐informed patient management decision. We recommend the use of bolus to cover the CIED during radiotherapy.

## CONFLICT OF INTEREST

The authors have no relevant conflict of interest to disclose.
